# Metagenomic Analysis Reveals a Nearly Complete Genome Sequence of *Alfalfa Mosaic Virus* from a Field Pea in Australia

**DOI:** 10.1128/MRA.00766-19

**Published:** 2019-08-01

**Authors:** S. Maina, L. Zheng, W. M. Kinoti, M. Aftab, N. Nancarrow, P. Trębicki, S. King, F. Constable, B. Rodoni

**Affiliations:** a Agriculture Victoria Research, Horsham, Victoria, Australia; b Agriculture Victoria Research, AgriBio, Bundoora, Victoria, Australia; Queens College

## Abstract

Here, we report the first nearly complete genome sequence of *Alfalfa mosaic virus* (AMV) obtained from a symptomatic field pea sample (Aus295) in Australia. Its genome RNA1 and RNA2 segments resembled those of the Argentinian isolate Manfredi, with 99.4% and 96.7% nucleotide (nt) identity, respectively; its RNA3 segment resembled that of Chinese isolate AMV-Gyn, with 99.6% nt identity.

## ANNOUNCEMENT

Alfalfa mosaic virus (AMV) is the type member of the genus Alfamovirus (family Bromoviridae) (1). It is a tripartite plus-sense single-stranded RNA (ssRNA) genome containing three segments, RNA1, RNA2, and RNA3 (2), packaged in a single virion. Globally, AMV is one of the most important plant viruses and has been found to occur in 305 species, 47 of which are dicot host species (3, 4). In Australia, AMV has been found in lucerne (Medicago sativa) and in pulses such as field pea (Pisum sativum) and faba bean (Vicia faba). It can be transmitted through seed, as well as by several aphid species, in a nonpersistent manner (5).

As part of a project to improve pulse seed diagnostic testing, 11 field pea leaf samples and 1 faba bean sample that were collected in different parts of Australia and had been preserved in CaCl_2_ were subjected to high-throughput sequencing for general virus detection. Total RNA was extracted from the 12 samples using a plant RNA miniprep kit (Zymo Research). The total RNA extracts were treated with RNase-free DNase (Invitrogen), and quality control checks were done as described previously (6). The 12 RNA sequencing (RNA-Seq) libraries were prepared using the TruSeq stranded total RNA sample preparation kit with the Ribo-Zero Plant (Illumina, San Diego, CA) method as described by Maina et al. (7) and Munguti et al. (8). The libraries were multiplexed in one lane and then subjected to MiSeq sequencing using a v3 kit (Illumina) with 2 × 251 cycles of paired-end reads.

Quality control of the fastq files was done using Trim Galore (9), with the minimum sequence length set to 50 bp and the minimum required adapter overlap (stringency) set to 1 bp. *De novo* assembly was performed using the metaSPAdes version 3.13.0 genome assembler (10) with default settings. The contigs of interest were imported in Geneious (11), and multiple alignment with reference sequences was performed using Muscle (12). Open reading frames were predicted and annotation made using the live annotate and predict command in Geneious (11), with transfer annotation selected and similarity set at 90%, while other settings were left as the default.

The sample Aus295 yielded 5,143,654 reads, and 5,125,062 reads remained after trimming. *De novo* assembly generated 3,753 contigs with 107,936 to 2,941,139 reads mapped to the contigs of interest and a coverage of 1,271 to 2,216×. The first Australian nearly full genome of AMV was obtained from a single field pea plant sample (Aus295) from New South Wales, Australia. Three contigs revealed AMV RNA genome segments (RNA1, RNA2, and RNA3) with GC contents of 42%, 43%, and 44%, respectively. RNA1 (3,753 nucleotides [nt]) encoded the P1 gene, RNA2 (2,579 nt) encoded the P2 gene, and RNA3 (2,033 nt) encoded the coat protein (CP) and movement protein genes, which is typical of the AMV genome (13, 14). A BLASTN search using BLAST+ v2.7 (15) revealed that Aus295 RNA1 and RNA2 genome segments closely resembled those of the Argentinian isolate Manfredi, with 99.4% and 96.7% nt identity, respectively (GenBank accession numbers KC881008 and KC881009), and its RNA3 closely resembled that of the Chinese isolate AMV-Gyn, with 99.6% nt identity (GenBank accession number MH332899). The high nucleotide match between the Aus295 and Manfredi RNA1 might reflect a reassortment event between the two isolates; however, a recombination study would be required to evaluate this deduction. The Aus295 RNA3 also had a 91.5% nt identity with the Australian AMV-N20 segment RNA3 (GenBank accession number AF332998) (16). Furthermore, the Aus295 CP gene was extracted and compared with eight other Australian AMV CP sequences. The Aus295 CP sequence closely resembled that of isolate Aq (GenBank accession number JX112758) from Western Australia (17), with 99.5% amino acid identity. This study forms part of a wider safer-seed certification project which aims to apply genomics and bioinformatics applications to increase the efficiency of current biosecurity procedures to safeguard the movement of pulse germplasm into and out of Australia, and the AMV genome information will be used to improve its detection in pulse crops using molecular methods.

### Data availability.

The sequences described here were deposited in GenBank under accession numbers LC485016 to LC485018. The raw data were deposited in the SRA under BioSample number SAMN11637366, which is part of BioProject PRJNA542720.
